# System Analysis of *MIRNAs* in Maize Internode Elongation

**DOI:** 10.3390/biom9090417

**Published:** 2019-08-27

**Authors:** Chuanxi Peng, Xing Wang, Tianyu Feng, Rui He, Mingcai Zhang, Zhaohu Li, Yuyi Zhou, Liusheng Duan

**Affiliations:** State Key Laboratory of Plant Physiology and Biochemistry, Engineering Research Center of Plant Growth Regulator, Ministry of Education & College of Agronomy and Biotechnology, China Agricultural University, No 2 Yuanmingyuan Xi Lu, Haidian District, Beijing 100193, China

**Keywords:** microRNA, internode elongation, dynamic expression, miRNA–mRNA network

## Abstract

MicroRNAs (miRNAs), the post-transcriptional gene regulators, are known to play an important role in plant development. The identification of differentially expressed miRNAs could better help us understand the post-transcriptional regulation that occurs during maize internode elongation. Accordingly, we compared the expression of *MIRNAs* between fixed internode and elongation internode samples and classified six differentially expressed *MIRNAs* as internode elongation-responsive miRNAs including *zma-MIR160c*, *zma-MIR164b*, *zma-MIR164c*, *zma-MIR168a*, *zma-MIR396f*, and *zma-MIR398b*, which target mRNAs supported by transcriptome sequencing. Functional enrichment analysis for predictive target genes showed that these miRNAs were involved in the development of internode elongation by regulating the genes respond to hormone signaling. To further reveal how miRNA affects internode elongation by affecting target genes, the miRNA–mRNA–PPI (protein and protein interaction) network was constructed to summarize the interaction of miRNAs and these target genes. Our results indicate that miRNAs regulate internode elongation in maize by targeting genes related to cell expansion, cell wall synthesis, transcription, and regulatory factors.

## 1. Introduction

The greatest improvements of maize (*Zea mays* L.) grain yield have been largely related to plant density and nitrogen fertilizer application [1,2]. However, excessive N fertilizer application and high planting density cause poor lodging resistance by forming weak basal internodes and increasing the height of the stem center of gravity [3,4]. Grain crop lodging includes stalk lodging and root lodging [5]. Stalk lodging is caused by bending or breaking of the lower internodes, while root lodging results from a failure in root soil integrity [6,7]. Plant height is an important character that determines the resistance of plants to stalk lodging [8]. Biologically, average internode length and internode number are the two main contributors of plant height and ear height, with average internode length being an important component of these traits [9]. In maize, stalk lodging positively correlates with the length of basal internodes [10,11]. Stalk lodging due to bending or breaking occurs most frequently at the third to fifth basal elongation internodes above ground, which is the seventh to ninth internodes of maize [12,13,14]. Therefore, it is of great significance to study the elongation mechanism of maize internodes, especially the seventh to ninth elongation internodes, for maize plant height regulation and lodging resistance cultivation.

Developmental stages of specific organs have been associated with different expression trends [15,16], epigenetic modifications [17], and posttranscriptional modification [18] at the molecular level. Numerous studies have shown that internode elongation can be altered by modifying the expression of a transcription factors that activate downstream target genes. Transcription factors related to internode elongation mainly include *ERFs*, *WRKYs*, and *TCPs*. Ethylene-response *AP2/ERF* factor *OsEATB* and *WRKY* transcription factor *OsWRKY78* regulate internode elongation by downregulating a gibberellin biosynthetic gene [19,20]. In addition, the *ERF11* transcription factor promotes internode elongation by activating gibberellin biosynthesis and signaling [21]. *AtTCP14* and *AtTCP15* influence internode length by promoting cell division in *Arabidopsis* [22]. MicroRNAs are approximately 21-nucleotide noncoding RNAs, which function through heterochromatin modification, post-transcriptional gene silencing, or translational inhibition, especially in epigenetic gene regulation [23]. miRNAs are widely found throughout the plant kingdom and highly conserved among plant species, including monocotyledons and dicotyledons [24]. Functional genomic studies have shown that mRNA cleavage and translational inhibition triggered by homology-mediated pairing of plant miRNAs with their mRNA targets are involved in a wide range of developmental processes [25,26,27,28]. In the past few years, knowledge about the roles of miRNAs in regulating stem development has increased significantly. Recent studies found that the defective expression of miR159 can also cause a decrease in plant height in rice [29]. The stunting and abnormal internode elongation of *Paulownia* were related to the expression level of *MIR164* [30]. miR166 mediated *ATHB15* mRNA cleavage is a principal mechanism for the regulation of vascular development in *Arabidopsis* inflorescence stems [31]. In rice, the expression of *OsMIR396d* affects cell elongation and division, thereby affecting internode elongation [32]. Furthermore, zma-miR169 and zma-miR396 play important roles in cell division and expansion in the internodes caused by hormone signals [32,33,34]. Moreover, OsmiR397 regulates its target gene, *OsLAC*, which is involved in the brassinosteroid sensitivity of the plants, leading to increases in internode length [35].

In summary, all these studies indicated that certain miRNAs play important regulatory roles in internode elongation. Although miRNAs associated with internode elongation have been extensively investigated in several plant species, to our knowledge, little information has been available to form a systematic view of miRNAs in maize internode elongation. Furthermore, stem lodging due to internode bending or rupture between seventh and ninth node causes 5%–25% of the annual loss of maize yield [12,13,14]. Therefore, it is essential to explore the molecular mechanisms of miRNAs involved in the regulation of basal internode elongation in maize. The main purposes of this study were to investigate the roles of miRNAs involved in the elongation of basal internodes above ground and to analyze the relationships of miRNAs and their target genes related to internode elongation. In this study, we generated and sequenced six maize RNA libraries from the seventh fixed and ninth elongating internodes on the third, fourth, or sixth day after the ninth spreading leaf. A whole-genome-wide identification of miRNAs was performed based on Solexa high-throughput sequencing technology for comprehensively understanding the changes of miRNAs expression patterns in the six samples and obtaining miRNAs related to internode elongation. Subsequently, we also analyzed the co-expression patterns between the expression profiles of miRNAs and their targets and constructed the miRNA–mRNA–PPI (protein–protein interaction) network to summarize the interaction of miRNAs and these targeted genes. Our studies contribute to understanding the possible roles of miRNAs and their targets in internode elongation of maize.

## 2. Materials and Methods

### 2.1. Plant Material, Growth Conditions, and Phenotypic Evaluation

The elite inbred line, ‘B73′ was used as the material in this study. Corn was grown in a glass greenhouse using soil culture. The growth conditions were 25 °C day temperature and 18 °C night temperature, accompanied by 16 h light and 8 h darkness. The materials were divided into two groups, one group was used for evaluating the length of seventh and ninth internode below the ear in four biological replicates. The internode length was measured from the ninth unrolled leaf for eight consecutive days. The other group was used as material for RNA deep sequencing. The sampling sites for seventh (seventh fixed node) and ninth (ninth elongating node) are respectively 4 mm in the 1 cm region at the top of the node, FR_7, and 4 mm in the 1–2 cm (elongation) region at the base, denoted as ER_9. Each part was sampled and repeated three times on the third, fourth, and sixth days after the ninth leaf was unrolled.

### 2.2. RNA Sequencing

Total RNA was purified from the 3, 4, and 6 d internode samples using Trizol reagent (Invitrogen) according to the manufacturer’s protocols. The concentration of RNA was measuring by Nanodrop2000 (NanoDrop Technologies) and the quality of RNA was determined by a 2100 Bioanalyzer (Agilent). When the quality of RNA was high (OD260/280 = 1.8~2.2, OD260/230 ≥ 2.0), the libraries were built. The samples of two internodes were selected for library construction, and 20 μg of total RNAs from each internode was supplied for Solexa deep sequencing. Libraries were sequenced with a read length of 100 bp (paired-end) and an insertion size of 300 bp on Illumina Genome analyzer (Majorbio, Beijing, China). SeqPrep (https://github.com/jstjohn/SeqPrep) and Sickle (https://github.com/najoshi/sickle) were used for the quality checks. Then, all high-quality reads were mapped to the B73 reference sequence (RefGen_v3) by using HISAT2 (http://ccb.jhu.edu/software/hisat2/index.shtml). The RNA-Seq data are deposited in Sequence Read Archive of the National Center for Biotechnology Information under the submission number SUB6197337.

### 2.3. Differential Gene Expression Analysis of miRNAs and mRNA

The frequency of miRNAs and mRNA was normalized as FPKM (fragments per kilobase per million reads) to correct copy numbers among different libraries. If the normalized read count of a given miRNA or mRNA was zero, the expression value was modified to 0.001 for further analysis. The fold change between the elongating internode and fixed internode was calculated as: Fold change = ER/FR. Additionally, |log2FC| ≥ 1 and P-adjust ≤ 0.05 were considered to be upregulated or downregulated in response to internode elongation, respectively. The P-value was calculated according to previously established methods [36]. Series test of cluster (STC) algorithm was used to analyze the dynamics of gene expression and observe the gene expression changes under different situations. The raw expression value was converted to log2 FPKM. Through clustering analysis of the expression trends of differentially expressed miRNA and mRNA in each group, the unique expression profiles were established.

### 2.4. Target Gene Prediction and Identification

PsRNATarget (http://plantgrn.noble.org/psRNATarget) was used to predict mRNA targets of plant miRNAs. The criteria for selection were allowing no more than three nucleotides mismatches, no more than one nucleotide base indels, and fewer than five G–U pairs of sequences. The predicted target genes were aligned with BLAST (http://blast.ncbi.nlm.nih.gov/) and annotated with Gene Ontology (GO) terms (http://www.geneontology.org/). The target genes were then mapped to the B73 maize genome (Version 2.0, http://www.maizegdb.org). To confirm the putative target of miRNA, the global changes was used to analyze the following data sets: Different miRNA expressed profiling (FR_7_3d versus ER_9_3d; FR_7_4d versus ER_9_4d; FR_7_6d versus ER_9_6d) and transcripts (FR_7_3d versus ER_9_3d; FR_7_4d versus ER_9_4d; FR_7_6d versus ER_9_6d). Target genes that were negatively correlated with miRNA expression were selected.

### 2.5. Function Enrichment Analysis

In order to evaluate the miRNA-gene regulatory network, the target sequences were annotated with agriGO software (http://systemsbiology.cau.edu.cn/agriGOv2/index.php) for specifying GO items to study the putative functions. The GO terms in the genes list were set against the background of all the genes in the genome. GO analysis was used to analyze the major functions of specific genes in the profiles of representative differentially expressed miRNA target genes. Only GOs that had a P-value of <0.05 were chosen. Enrichment provides a measure of the significance of the function: As the enrichment increases, the corresponding functions are more specific, which helps us to find GOs with more specific function descriptions in the experiment.

### 2.6. Construction of miRNA–mRNA and PPI Networks

mRNA was selected based on the prediction of miRNA target genes and the principle that the expression pattern of mRNA was negatively correlated with miRNA from expression profiles. Furthermore, combining the differentially expressed miRNAs and mRNAs, a core miRNA–mRNA regulatory network was constructed by using the Cytoscape software. In the miRNA–mRNA network, the relationship between miRNA and mRNA was represented by an edge. In order to further analyze the interaction between miRNA target genes, the identified target genes were used for PPI (protein–protein interaction) analysis. The protein names were obtained on Uniprot (https://www.uniprot.org) and NCBI (https://www.ncbi.nlm.nih.gov/protein/). ‘The relationship between proteins is realized through STRING: Function protein association networks (https://string-db.org). Then, a protein–protein interaction network was constructed by using the Cytoscape software. The thickness of the edges was adjusted from the combined score. The size of nodes was determined by the count of the interactions.

## 3. Results

### 3.1. Development of Internodes

**Figure 1:**
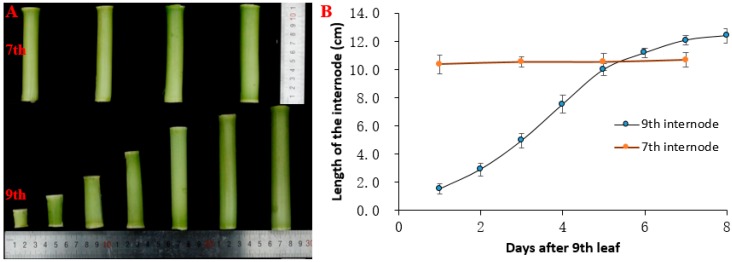
Changes in the length of the seventh and ninth internodes. (**A**) Phenotypes of seventh and ninth internodes length in B73. The bottom half shows the phenotype of the ninth internode from day 1 to day 7 after nine leaves of maize were unfolded. The top half shows the phenotype of the seventh internode on day 1, 3, 5, and 7 after nine leaves of maize were unfolded. (**B**) Elongation curves of the seventh and ninth internodes after nine leaves of maize were unfolded. The blue dots from left to right indicate the length of the ninth internode from day 1 to day 8. The orange dots from left to right indicate the length of the seventh internode on day 1, 3, 5, and 7 after nine leaves of maize were unfolded. Values are means ± SE of four biological replicates. The data are presented in Supplementary Table S1.

After the ninth leaf was expanded, the length of the seventh internode did not change significantly at the time points (1, 3, 5, and 7 d). The length of the seventh internode was about 10.5 cm. The elongation trend of the ninth internode presented an s-shaped curve with the length of the ninth internode continuously increasing. The final length of the ninth internode was approximately 12.4 cm. The elongation rate first increased and then decreased, and the internode elongation rate of three to six days was higher, among which the maximum elongation was on the fourth day (Figure 1B).

### 3.2. Identification of miRNAs Associated with Internode Elongation

**Figure 2:**
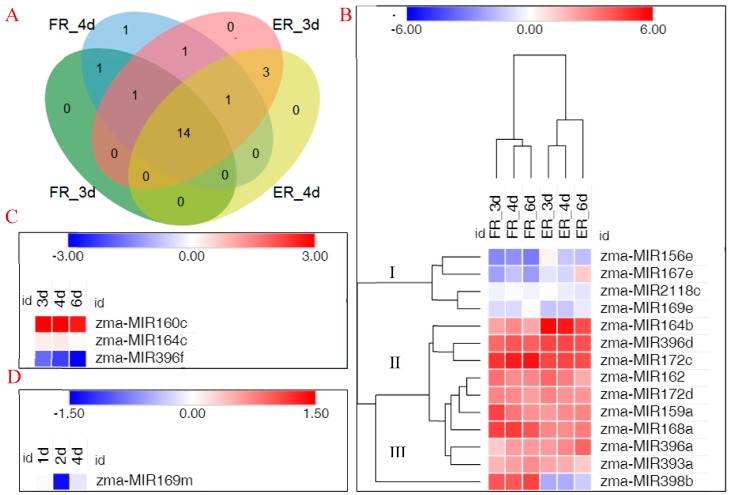
Distribution of microRNAs (miRNAs) at different stages of internode elongation. Comparison of miRNAs identified between fixed internode region and elongation area. (**A**) Venn diagram of miRNAs associated with internode elongation at FR and ER after nine leaves of maize were unfolded. The 1 cm region of seventh fixed node top and the 1 cm (elongation) region of ninth elongating node base are denoted as FR and ER, respectively. (**B**) Temporal patterns of microRNA expression in both fixed and elongating internode samples. (**C**) miRNAs specifically expressed in the elongating internode samples; (**D**) microRNAs specifically expressed in the fixed internode samples. The color bars show log2 FPKM (fragments per kilobase per million reads). FR, seventh fixed internode region; ER, ninth elongating internode region; 3d, 4d, or 6d, the third, fourth, or sixth day after the ninth spreading leaf. The data are presented in Supplementary Tables S2 and S3.

In order to describe the expression pattern of *MIRNAs* in the process of internode elongation, transcriptome sequencing was performed on samples taken at different time points, which were in a high internode elongation rate. Thus, we selected 3, 4, and 6 d internode samples to construct RNA libraries. Six total RNA libraries were built from the 1 cm region of seventh fixed node top and the 1 cm elongation region of ninth elongating internode base, with three sample repeats for each treatment. Through homologous analysis, we identified known miRNAs following the selection criteria for miRNA sequences with a length of at least 18 nt, and there were at most two mismatches compared with all known plant miRNA sequences in miRBase 22.1. Among these known miRNAs, more than 14 miRNAs were common across the four libraries, and less than three miRNAs were specific to internode elongation (Figure 2A). For example, *zma-MIR160c*, *zma-MIR164c*, and *zma-MIR396f* were expressed only in elongating internode region samples, while *zma-MIR169m* was expressed only in the fixed internode samples. In addition, the internode sample on the third day after the ninth spreading leaf shared two miRNA families that did not occur in the fourth day in elongating internode region, *zma-MIR159f* and *zma-MIR319d*. *Zma-MIR164d* was specifically expressed in the fourth day fixed internode library.

Among the identified miRNAs, 22 known miRNAs were used to analyze differential expression during internode elongation. Fourteen miRNAs were identified by comparing the expression profiles, which were differentially expressed between the fixed and elongating internode samples. Based on the expression patterns of 14 known miRNAs during internode elongation, three clusters were constructed by cluster analyses (Figure 2B). Cluster I and Cluster II contained the same expression pattern, showing a lower or higher expression, both at fixed and elongating internodes; Cluster III contained miRNAs with opposite expression patterns at fixed and elongating internodes. Three microRNAs were expressed only in elongating internode samples, which show the same expression pattern in the process of internode elongation (Figure 2C). *Zma-MIR169m* was expressed only at fixed internode, while it presented a change of expression level in different time processes (Figure 2D).

### 3.3. Comprehensive Analysis for Predictive Target Genes of miRNA

**Figure 3:**
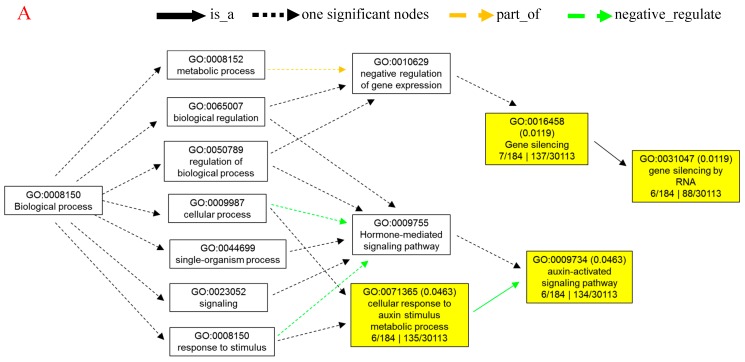

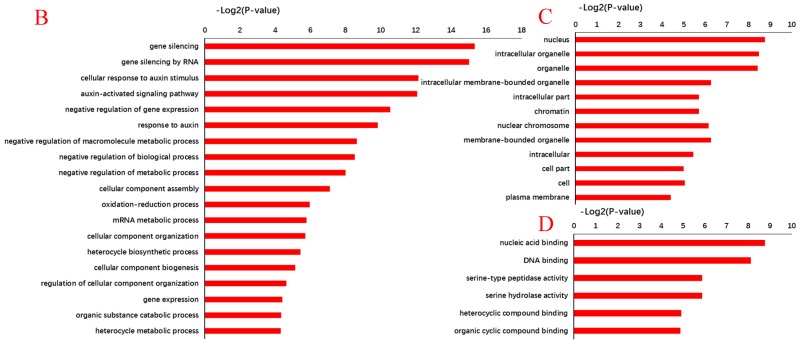
Gene ontology (GO) analysis for predictive targeted genes of differentially expressed miRNAs associated with internode elongation. Gene ontology enrichment analysis of biological process (**A**). −Log2 (P-value) of the corresponding biological process (**B**), cellular component (**C**), and molecular function (**D**). Log2 (P-value) is the negative logarithm of P-value; a bigger Log2 (P-value) indicates a smaller P-value. Detailed data are presented in Supplementary Tables S4 and S5.

To investigate the relationship between differentially expressed miRNAs and internode elongation, gene ontology (GO) analyses were applied. *MIR160c, MIR164b, MIR164c, MIR168a, MIR396f*, and *MIR398b* were selected, which had showed extremely significant differential expression in elongated and fixed regions at the different time points (Figure 2). Based on target prediction, more than 200 genes were identified as the potential target genes of these six miRNAs. GO analysis of representative profiles of miRNA putative target genes is shown in Figure 3. Gene ontology enrichment analysis of biological process revealed that miRNA can participate in a single-organism cellular biological process through gene silencing by RNA and auxin signaling pathway. miRNAs could negatively regulate gene expression through RNA-induced gene silencing, thereby regulating biological and metabolic processes. More importantly, miRNAs regulated cellular and single-organism processes by mediating cell responses to auxin stimulus metabolic process via auxin activated signaling pathways (Figure 3A). GO terms were mainly related to regulation of gene expression, signaling pathway, metabolism, and development associated process, such as gene silencing, auxin-activated signaling pathway, and cellular response to auxin stimulus on the biological process level (Figure 3B). On the cellular component level, GO terms mainly included intracellular organelle, nucleus, and intracellular membrane-bound organelle (Figure 3C). On the molecular function level, GO terms were mainly involved in nucleic acid binding, DNA binding, protein binding, and enzyme activity (Figure 3D).

### 3.4. Identification and Analysis for Target Genes of Internode Elongation Related miRNA

**Figure 4:**
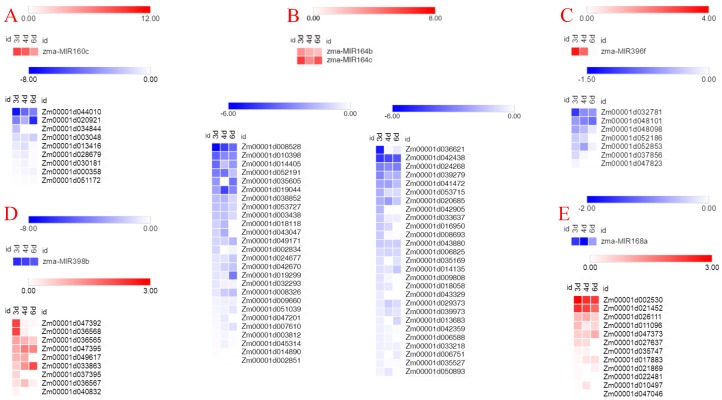
Co-expression patterns of microRNAs and their targets. (**A**–**E**) shows the expression profiles of different microRNA–mRNA pairs. The color bars show log2-fold change value between elongation region and fixed internode samples. The data are presented in Supplementary Table S6.

The global changes in the expression patterns of miRNA and mRNA during internode elongation were compared in maize. As we all known, miRNA leads to downregulation of target gene expression at the transcriptional or translation level [37]. Negative correlation between miRNA and its target genes has been reported in the literature. By conjunction with the predicted miRNA targets, further analyzing and integrating expression profiles of microRNA and mRNA, we identified six miRNA–mRNA sets potentially involved in the regulation of internode elongation following the negative correlation. miRNA and mRNA expression profiling analysis confirmed the inverse expression patterns between miRNAs and their target mRNAs, with the exception of very few target genes (Figure 4). In the process of internode elongation, *MIR160c, MIR164b, MIR164c*, and *MIR396f* were dramatically induced. With the gradual termination of internode elongation, the upregulated levels of the four miRNAs were gradually decreased. Their target mRNAs showed the expected negative regulation pattern. To our surprise, *Zm00001d020921, Zm00001d003048, Zm00001d000358, Zm00001d035605, Zm00001d019299, Zm00001d019044, Zm00001d048101*, and *Zm00001d003048* showed no remission of inhibition or even decreased expression level with the decrease of miRNA expression level (Figure 4A–C). During the internode elongation, the inhibition of the expression levels of *MIR398b* and *MIR168a* is attenuated over time. Consistent with this, the upregulation levels of most target genes are gradually decreasing. Interestingly, *Zm00001d047395, Zm00001d033863*, and *Zm00001d047373* were not inhibited with the increase of miRNA expression level (Figure 4D,E). The interaction between proteins of target genes may affect the regulation of miRNAs on the expression level of target genes.

**Figure 5:**
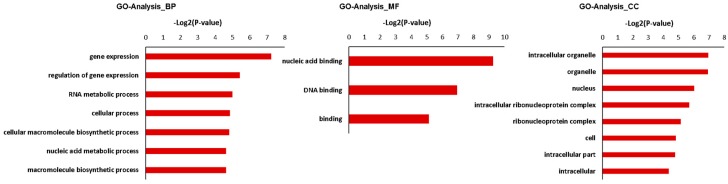
GO analysis for targeted genes of representative differentially expressed miRNA. −Log2 (P-value) of the corresponding biological process (BP), molecular function (MF), and cellular component (CC). Log2 (P-value) is the negative logarithm of P-value; a bigger Log2 (P-value) indicates a smaller P-value. The data are presented in Supplementary Tables S5 and S6.

GO analysis was performed on target genes of internode elongation-related miRNAs. GO analysis of target genes identified by miRNA and mRNA transcriptome is shown in Figure 5. The target genes of the differentially expressed miRNAs were classified into 18 categories: seven biological processes, three molecular functions, and eight cell component categories. They were mainly related to regulation of gene expression, metabolism, and development-associated process, such as gene silencing, cellular process, and cellular macromolecule biosynthetic process on the biological process level. Within the molecular function category, GO terms were mainly involved in nucleic acid binding and DNA binding. On the cellular component level, GO terms mainly included intracellular organelle, nucleus, and organelle (Figure 5).

### 3.5. Analysis of the miRNA–mRNA Network and Construction of PPI

**Figure 6:**
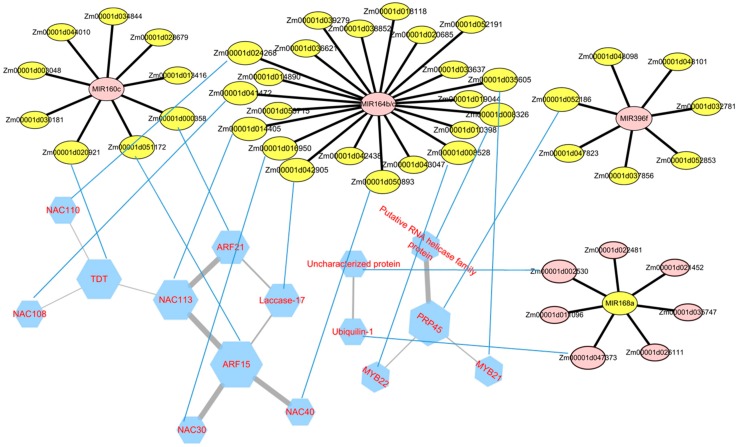
The biomolecular networks for miRNA–mRNA and STRING protein interaction (between the genes, the specific interactive mode will not be discussed here). The network for miRNAs and their target mRNA and their relationship is represented by a black edge. The pink circles mean upregulated, and the yellow circles mean downregulated. The blue hexagon represents the protein translated by the target gene. The thickness of the edges is adjusted from the gradient of the combined score. The size of nodes is determined by the count of the interactions. The mRNA and the protein are connected by blue lines. (Figure 6 is designed using open source software STRING and Cytoscape 3.4.0). More details can be found in Supplementary Table S7.

Based on these miRNAs and their targeted genes, the miRNA–mRNA network was constructed to summarize the interaction of miRNAs and these targeted genes. As shown in Figure 6, these upregulated miRNAs that had higher degree were in the center of the network. For example, ZmNAC30 *(Zm00001d016950)*, ZmNAC108 *(Zm00001d041472)*, ZmNAC113 *(Zm00001d014405)*, ZmMYB22 *(Zm00001d008528)*, Uncharacterized protein (*Zm00001d018118*), Harpin-induced protein (*Zm00001d039279*), Cupredoxin superfamily protein (*Zm00001d052191*), and laccase-17 (*Zm00001d042905*) are the presumed targets of *zma-MIR164b/c*. The network of four upregulated miRNAs, including *zma-MIR160c, zma-MIR164b, zma-MIR164c*, and *zma-MIR396f*, may repress the gene expression of transcription factors, for example, *WRKY30* (*Zm00001d044010*), *MYB22* (*Zm00001d008528*), *NAC30* (*Zm00001d016950*), and *NAC113* (*Zm00001d014405*) respectively. In addition, the decreased miRNAs including *zma-MIR168a* and *zma-MIR398b* lead to the increased expression of their target genes *bZIP* transcription factor to regulate related signal transduction.

In order to further analyze how miRNA affects internode elongation by affecting target genes, PPI (protein and protein interaction) analysis was performed on identified target genes. As shown in Figure 6, ARF15 (*Zm00001d051172*) interacts with four proteins which are the target genes of *zma-MIR164b/c*, named ZmNAC113 (*Zm00001d014405*), ZmNAC30 (*Zm00001d016950*), ZmNAC40 (*Zm00001d050893*), and laccase-17 (*Zm00001d042905*). TDT (tonoplast dicarboxylate transporter, *Zm00001d016950*), NAC113 *(Zm00001d014405)*, and PRP45 (Pre-mRNA-splicing factor prp45, *Zm00001d052186*) could interact with each other. *ZmNAC108* (*Zm00001d041472*) and *ZmNAC110* (*Zm00001d024268*) are target genes of *zma-MIR164 b/c* too. They could interact with TDT protein (*Zm00001d016950*). Putative RNA helicase family protein (*Zm00001d008326*), MYB21 (*Zm00001d035605*), and MYB22 (*Zm00001d008528*) are the interacting proteins of PRP45 (*Zm00001d052186*), and they are all the target genes of *zma-MIR164b*. Unknown protein (Hypothetical protein, *Zm00001d002530*) and Ubiquilin-1 (Ubiquitin domain-containing protein DSK2b, *Zm00001d047373*), both targets of *zma-MIR168a*, are interacting proteins. Different target genes of certain microRNA tend to have common interacting proteins, which may be caused by the resemblances of protein sequences caused by the high similarity of sequences within the different target genes. miRNA and target sequences have extensive complementarity. This can be explained by the wide complementarity of miRNA with target sequences and the tendency of miRNA to act on multiple members of a protein family.

## 4. Discussion

### 4.1. miRNAs Involved in Maize Internode Elongation

miRNAs, the post-transcriptional gene regulators, are known to play an important role in the process of growth and development. The identification of differentially expressed miRNAs could better help us understand the post-transcriptional regulation that occurs during internode elongation. Accordingly, we compared the expression of miRNAs between fixed internode and elongation internode samples and classified six differentially expressed miRNAs as internode elongation-responsive miRNAs, which target mRNAs supported by transcriptome sequencing. *zma-MIR160c, zma-MIR164b, zma-MIR164c*, and *zma-MIR396f* were upregulated in the development of internodes under maize ear. In contrast, *zma-MIR168a* and *zma-MIR398b* were downregulated during internode elongation (Figure 2). Target prediction and functional annotation of the significantly differentially expressed microRNAs recognized microRNAs potentially associated with plastic internode elongation in maize (Figure 3). Identification and analysis for target genes of internode elongation related miRNA further confirmed that miRNA could participate in internode elongation of maize (Figure 4 and Figure 5). Similar results have previously been observed in other species. For example, zma-miR160 can affect the development of the lower internode of maize ear through hormone signal transduction [38]. In addition, the expression of zma-miR168 was associated with heterosis of internode elongation in hybrids [34]. The stunting and abnormal internode elongation of *Paulownia* were related to the expression level of miR164 [30]. In rice, the expression of *OsMIR396d* affects cell elongation and division, thereby affecting internode elongation [32]. Furthermore, the silencing of OsmiR398a leads to the phenotype of significantly shortened internodes in rice [39]. These results indicate that the miRNAs in maize inbred line B73 were similar to other species during internode elongation.

**Figure 7:**
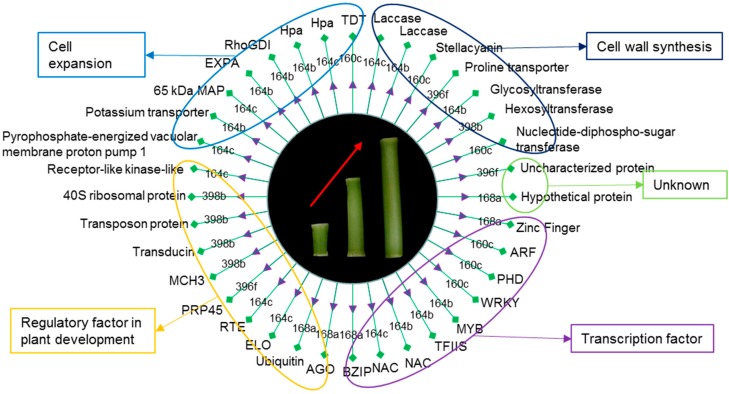
A proposed miRNA-regulatory network during internode elongation of maize.

The regulation of miRNA on mRNA expression is mainly achieved through the splicing of the target mRNA or repression of target protein translation. In the present study, the target genes of the screened miRNA target genes related to internode elongation were mostly the transcriptional and translational regulation factors that may affect global changes in gene expression. Transcription factors regulated by miRNAs may be crucial for internode elongation, which accounts for one-third of miRNA target genes. In addition to transcription factors, we also found that miRNAs target kinase, helicase, oxidoreductase, transferase, transporter, and protein-coding genes that play roles in the progress of internode elongation (Figure 7).

### 4.2. miRNAs Affect Cell Expansion and Cell Wall Synthesis by Regulating Their Targets in Maize

Previous studies have demonstrated that *TDT* (tonoplast dicarboxylate transporter) is critical for the regulation of pH homeostasis under altered pH conditions [40]. pH levels are necessary for dilatation protein activity [41]. Cell wall acidification resulted in upregulation of the protein abundance and gene expression of dilation, which was critical for internode growth due to cell elongation [40,42,43]. In this study, *ZmTDT* (*Zm00001d020921*) was inferred to be a target gene of *zma-MIR160c*, which means that *zma-MIR160c* may affect cell pH and thus control internode development by regulating the expression of *ZmTDT*. In addition, potassium is used as a major active solute to maintain turgor and to drive irreversible and reversible changes in cell volume. K^+^ concentration gradient caused by the potassium (K^+^) transporter can lead to a change in cell volume [44]. Moreover, EXPA1 is an active dilatation protein and its expression is associated with internode elongation [45,46]. It is possible that *zma-MIR164b* mediates cell expansion by targeting potassium (K^+^) transporter (*Zm00001d036621*) and affecting the expression of *ZmEXPA1* (Z*m00001d043047*, Figure 4).

The reduced expression of *OsRhoGDI2* conferred hypersensitivity to gibberellin (GA) stress in rice [47]. Therefore, in the process of internode elongation, the highly expressed *zma-MIR164b* may improve the sensitivity to gibberellin by inhibiting the expression of *ZmRhoGDI1* (*Zm00001d010398*, Figure 4). In addition, miR164b regulates *Hpa1*-induced plant growth enhancement and associated physiological and molecular responses. The harpin protein, Hpa1, induces a variety of growth-promoting responses in rice, activating the ethylene and gibberellin signaling pathways, improving photosynthesis rates and *EXPANSIN* (*EXP*) gene expression levels, and thereby promoting vegetative growth [48]. Previous studies have found that 65 kDa MAP is involved in the elongation growth of azuki bean epicotyls [49]. It is possible that *zma-MIR164c* targets 65 kDa MAP (*Zm00001d020685*) to regulate plant microtubules, thus leading to the elongation of plant cells.

In order to maintain the integrity of the wall and to adjust its properties to adapt to the changing needs of the cell, plants respond to the growth of the stem by remodeling matrix polysaccharides and by regulating the cell wall biosynthetic machinery. Hybrid proline-rich proteins (HyPRPs) are crucial players in cell elongation. Proline transporter 2 (*Zm00001d042438*), the putative target gene of zma-miR164c, plays an important role in plant development by providing proline as a source of nitrogen and energy and a component of plant cell wall proteins, which is required for lignifications, xylem differentiation, and cell wall modification during plant development [50]. Meanwhile, Nucleotide-diphospho-sugar transferase (*Zm00001d034844*, the *zma-MIR160c* putative target gene), glycosyltransferase (*Zm00001d053715*, the *zma-MIR164b* putative target gene), and hexosyltransferase (*Zm00001d049617*, the *zma-MIR398b* putative target gene) affect the cell wall development of elongated internodes by participating in the transition from primary cell wall to secondary cell wall synthesis [51]. Laccases-17 (*Zm00001d042905*) are correlated with lignin biosynthesis in *Arabidopsis* and *Zinnia* stem tissues [52,53]. Furthermore, *OsLAC* was found to be involved in the sensitivity of plants to brassinosteroids [35]. This suggests that *zma-MIR164b* may affect the synthesis of lignin during internode development by acting on Laccase (*Zm00001d042905*). Stellacyanin, a germin-like protein, was described an oxalate oxidase, strongly associated with hemicelluloses, the synthesis of which is linked to the increase in cell wall extensibility [54]. *zma-MIR396f* may target stellacyanin (*Zm00001d048101*) to affect cell fibrogenesis during internode elongation [55].

### 4.3. miRNAs Targeting Transcription and Regulatory Factors Are Involved in Internode Elongation

Half of the targets were found to be transcription factors (TFs) and regulators of plant development. These include auxin-responsive genes, members of the *WRKY* transcription factor family, the *MYB* transcription factor family, the *NAC* transcription factor family, *ARF* transcription factor family, *bZip* transcription factor family, the transcription elongation factor (*TFIIS*) family, the zinc finger-like family and regulators (Figure 7).

In order to adapt to the changing needs of cells, plants regulate the biosynthesis mechanism of cell walls in response to stem growth. *zma-MIR164c* could affect the dynamic properties of cell walls by acting on regulatory factor *RLKL* (receptor-like kinase-like, *Zm00001d019044*) that regulate cell wall function [56,57]. Elongator mutants (abo1/elo2/elp1, elp2, elo3/elp3, and elo1/elp4) have reduced root growth, abscisic acid hypersensitivity, and an increased accumulation of anthocyanins [58,59]. *zma-MIR164c* may affect the sensitivity to ABA by acting on *ELO1* (elongation defective-like1, *Zm00001d033637*), thus affecting the internode elongation. Previous findings demonstrate that *RTE1* (REVERSION-TO-ETHYLENE SENSITIVITY1, *Zm00001d038852*) is a negative regulator of ethylene signaling, which means *zma-MIR164c* may affect internode elongation by ethylene signaling [60]. Moreover, it has been found that *cry1* (cryptochrome 1) mediates photoreceptor signaling networks in plant responses to shade [61]. This means *zma-MIR398b* may be involved in stem elongation by regulating *ZmMCH3* (maize CRY1 homolog3, *Zm00001d036565*). Furthermore, *ZmAGO1* (*Zm00001d011096*), a negative feedback regulator of the RNA-induced silencing complex (RISC), was predominately repressed in the elongating internode of the B73 [62]. Furthermore, *ZmPRP45* (*Zm00001d052186*), a *zma-MIR396f putative* target gene, is spliceosome associated throughout the splicing process and is essential for pre-mRNA splicing [63]. In addition, transducin related to protein targeting was upregulated due to downregulation of *zma-MIR398b* (*Zm00001d047392*) and plays an important role in the internode elongation [64].

WRKY proteins are a large super family of transcriptional regulators primarily involved in various plant physiological programs. *GhWRKY15* was greater in the stems compared with the expression in the cotyledon of cotton, and the stems of transgenic tobacco displayed faster elongation compared with wild-type plants [65]. Moreover, the WRKY transcription factor regulates stem elongation in rice [20]. The conserved function of MlWRKY12 existing in secondary cell wall formation of monocotyledonous species has been revealed [66]. It is possible that expression of *zma-MIR160c*, which acts on the WRKY transcription factor (WRKY74, *Zm00001d044010*), enhances cell division in the internodes.

*ARFs* are the important *TFs* in the auxin signaling pathway, regulating the transcription of auxin-responsive genes. *AtARF10*, *AtARF16*, and *AtARF17* in *Arabidopsis* are the targets of miR160 [67]. In addition, genome-wide analyses of target genes of an auxin response factor (ARF6) that regulates hypocotyl elongation have also been done [68]. In the present work, *zma-MIR160c* were validated to be upregulated in ninth elongating internode. It seemed to play a negative regulation role in regulating internode elongation of basal elongation internodes above ground.

Major functions of *MYB* are regulated by miRNA include primary and secondary metabolism, cell fate and identity, developmental processes, etc. [69]. Previous studies have shown that *MYBs* are targeted by miR159, miR828, and miR858 in *Arabidopsis* and *Gossypium hirsutum* [70,71]. Functional deletion mutation in *OsGAMYB* leads to internode elongation [72]. This may be related to the regulation of *MYB21* by gibberellin [73]. *NAC1* is induced by auxin and acts downstream of *TIR1* [74]. In addition, a gibberellin-mediated DELLA-NAC signaling cascade regulates cellulose synthesis in rice [75]. Furthermore, zma-miR164 was dominantly repressed in the hybrid, which indicated that *NAC* transcription might be upregulated and result in enhancement of auxin and GAs signals, followed by the expansion of internodes [34]. In the current work, *zma-MIR164* was upregulated at the ninth internode, which may be related to tissue specificity and plant materials.

## 5. Conclusions and Prospects

In conclusion, we compared the expression of miRNAs between fixed internode and elongation internode samples and classified six differentially expressed miRNAs as internode elongation-responsive miRNAs. These miRNAs might regulate the internode elongation and development by their targets associated with transcription factor, regulatory factor in plant development, cell expansion, and cell wall synthesis. The miRNA–mRNA–PPI (protein and protein interaction) network was constructed to summarize the interaction of miRNAs and these targeted genes to further reveal how miRNA affects internode elongation by affecting target genes. The involvement of miRNAs and their targets contribute to understanding the possible roles of miRNAs in internode elongation of maize.

However, our understanding of mechanism through which the miRNAs regulate internode elongation in maize is just beginning. The main miRNA–mRNA interactions, identified here to have a role in regulating internode elongation, require further investigation. Identification of target gene splicing sites by miRNA is imperative. In addition, it is necessary to detect the tissue expression sites of miRNA and target genes by in situ hybridization. STRING protein interactions have found that several target genes of certain microRNA tend to have common interacting proteins, which need to be further verified. Furthermore, the use of transgenic and gene-editing methods to alter the expression levels of internode elongation-related miRNAs will contribute to their functional identification. This provides a valuable reference for future functional analysis.

## Supplementary Materials

The following are available online at https://www.mdpi.com/2218-273X/9/9/417/s1, Table S1: Changes in the length of the seventh and ninth internodes; Table S2: Distribution of miRNAs at different stages of internode elongation; Table S3: Relative expression of miRNAs identified in this study; Table S4: Predictive target genes of differentially expressed miRNAs; Table S5: GO analysis for predictive targeted genes of differentially expressed miRNAs; Table S6: Identification and analysis for target genes of internode elongation related miRNA; Table S7: Detailed information of miRNA-mRNA-PPI network.
